# Reno-protective effects of perioperative dexmedetomidine in kidney transplantation: a systematic review and meta-analysis of randomized controlled trials

**DOI:** 10.1007/s11255-023-03568-3

**Published:** 2023-03-30

**Authors:** Mohamed T. Abuelazm, Ahmed Ghanem, Amit Johanis, Abdelrahman Mahmoud, Abdul Rhman Hassan, Basant E. Katamesh, Mostafa Atef Amin, Basel Abdelazeem

**Affiliations:** 1 Faculty of Medicine, Tanta University, Tanta, Egypt; 2 Cardiology Department, The Lundquist Institute, Torrance, CA USA; 3 Faculty of Medicine, Creighton University, Phoenix, AZ USA; 4 Faculty of Medicine, Minia University, Minia, Egypt; 5 Faculty of Medicine, Cairo University, Cairo, Egypt; 6 Department of Internal Medicine, McLaren Health Care, Flint, MI USA; 7 Department of Internal Medicine, Michigan State University, East Lansing, MI USA

**Keywords:** DEX, Dexmedetomidine, Kidney transplantation, Systematic review, Meta-analysis

## Abstract

**Background and objective:**

There is currently no FDA-approved medical therapy for delayed graft function (DGF). Dexmedetomidine (DEX) has multiple reno-protective effects preventing ischemic reperfusion injury, DGF, and acute kidney injury. Therefore, we aimed to evaluate the reno-protective effects of perioperative DEX during renal transplantation.

**Methods:**

A systematic review and meta-analysis synthesizing randomized controlled trials (RCTs) from WOS, SCOPUS, EMBASE, PubMed, and CENTRAL until June 8th, 2022. We used the risk ratio (RR) for dichotomous outcomes and the mean difference for continuous outcomes; both presented with the corresponding 95% confidence interval (CI). We registered our protocol in PROSPERO with ID: CRD42022338898.

**Results:**

We included four RCTs with 339 patients. Pooled risk ratio found no difference between DEX and placebo in reducing DGF (RR: 0.58 with 95% CI [0.34, 1.01], p = 0.05) and acute rejection (RR: 0.88 with 95% CI [0.52, 1.49], p = 0.63). However, DEX improved short-term creatinine on day 1 (MD: − 0.76 with 95% CI [− 1.23, − 0.3], p = 0.001) and day 2 (MD: − 0.28 with 95% CI [− 0.5, − 0.07], p = 0.01); and blood urea nitrogen on day 2 (MD: − 10.16 with 95% CI [− 17.21, − 3.10], p = 0.005) and day 3 (MD: − 6.72 with 95% CI [− 12.85, − 0.58], p = 0.03).

**Conclusion:**

Although there is no difference between DEX and placebo regarding reducing DGF and acute rejection after kidney transplantation, there may be some evidence that it has reno-protective benefits because we found statistically significant improvement in the short-term serum creatinine and blood urea nitrogen levels. More trials are required to investigate the long-term reno-protective effects of DEX.

**Supplementary Information:**

The online version contains supplementary material available at 10.1007/s11255-023-03568-3.

## Introduction

End-Stage Renal Disease (ESRD) is a global health burden. In the 2021 annual report, the United States Renal Data System (USRDS) reported a continuous increase in the ESRD prevalence over the years, with 2302 cases per million in 2019 compared to 1582 cases per million in 2000 [1]. Dialysis and kidney transplantation are the only treatment options for ESRD. Transplantation is superior to dialysis in every aspect. With the increase in ESRD cases, the Kidney transplantation rate increased from 15,220 in 2000 to 24,502 in 2019 [2]. Patients receiving kidney transplants are at a greater risk for graft ischemia–reperfusion injury (IRI), which decreases the rates of transplantation success [3, 4].

The transplanted kidney suffers from ischemia and a lack of nutrients during renal transplantation. Ischemia starts with sympathetic overactivity from the surgical stress causing vasoconstriction of the renal arteries [5]. Moreover, transplanting kidneys from deceased donors, clamping the renal arteries, and prolonging the time interval between extraction, transport, and implantation can further make the kidney more vulnerable to ischemic effects [5]. On restoring the blood flow after transplantation, the graft becomes at risk of oxidative injury and inflammation, damaging the tubular and endothelial cells, and eventually leading to IRI [4]. IRI can lead to delayed graft function (DGF), reduced graft survival, and acute kidney rejection [6–8].

Dexmedetomidine (DEX) is an alpha-2-adrenoreceptor agonist [9] that has procedural sedative, analgesic, sedative, anxiolytic, and sympatholytic effects [10, 11]. DEX downregulated the inflammatory reactions in rats [12]. Furthermore, in the meta-analysis conducted by Wang et al. [13] over 60–70 studies in surgical patients, they found that DEX attenuated perioperative stress, inflammation, and immune reactions compared to the control group [13]. Yang et al. [14] and Li et al. [15] reported that DEX reduced the activation of NLRP3 inflammasome. Regarding renal effects, on one hand, DEX was also reported to have a reno-protective effect against IRI and Acute Kidney Injury (AKI) [16–21] as well as DGF [22]. On the other hand, DEX might cause dose-dependent bradycardia and hypotension [23], which might adversely affect renal microcirculation [23, 24].

Despite the growing evidence about the reno-protective effects of DEX, its perioperative use in kidney transplantation is still inconclusive, with multiple recent trials investigating it [25–28]. Therefore, we performed this systematic review and meta-analysis to synthesize evidence from the published randomized controlled trials (RCTs) on the reno-protective efficacy of perioperative DEX in patients undergoing kidney transplantation.

## Methodology

### Protocol registration

Our review procedure was registered and published in PROSPERO with ID: CRD42022338898. We conducted a systematic review and meta-analysis sincerely guided by the Preferred Reporting Items for Systematic Reviews and Meta-Analyses (PRISMA) statement [29] and the Cochrane Handbook of Systematic reviews and meta-analysis [30]. The PRISMA 2020 checklist is illustrated in Table S1.

### Data sources and search strategy

Web of Science, SCOPUS, EMBASE, PubMed (MEDLINE), and Cochrane Central Register of Controlled Trials (CENTRAL) were systematically searched by two reviewers (B.A. and M.T.) from inception until June 8th, 2022. No search filters were used. The detailed search approach and results are outlined in Table S2.

### Eligibility criteria

We included RCTs with the following PICO criteria: population (P): patients receiving either living or deceased-donor kidney transplant; intervention (I): perioperative DEX regardless of dosage and duration of administration; control (C): saline placebo; outcomes (O): primary outcomes: incidence of DGF defined as required dialysis within one week following transplantation [31, 32] and incidence of acute graft rejection. Our secondary outcomes are the post-transplant kidney function tests irrespective of the postoperative day (POD) of assessment (creatinine, cystatin, blood urea nitrogen (BUN), urine output, and glomerular filtration rate (GFR).

Animal studies, pilot studies, observational studies (cohort, case–control, cross-sectional, case series, and case reports), single-arm clinical trials, in vitro studies (tissue and culture studies), book chapters, editorials, press articles, and conference abstracts were all excluded from our analysis.

### Study selection

After duplicates were removed using Covidence online software, two investigators (A.H. and A.M.) independently evaluated the titles and abstracts of the retrieved records. (5) Then, they checked the full texts of the relevant records for the previously mentioned eligibility criteria. To resolve any disagreements, a third reviewer (B.K.) was invited.

### Data extraction

Using a pilot-tested extraction form, four reviewers (A.H., A.M., B.K., and M.A.A.) independently extracted the following data from the included articles: study characteristics (first author name, year of publication, country, study design, total participants, DEX’s dose and duration of administration, donor status, and follow-up duration); baseline information (age, sex, body mass index, hypertension, diabetes, dialysis history, ABO incompatibility, serum creatinine, and heart rate); efficacy outcomes data (incidence of DGF, acute rejection, and post-transplantation kidney function tests). Disagreements were resolved through discussion.

### Risk of bias and quality assessment

Using The Cochrane Collaboration's technique for assessing the risk of bias in randomized trials, four reviewers (A.H., A.M., B.K., and M.A.A.) independently assessed the included studies for risk of bias (ROB) [33], based on the following domains: random sequence generation (selection bias), allocation concealment (selection bias), blinding of participants and personnel (performance bias), blinding of outcome assessment (detection bias), incomplete outcome data (attrition bias), selective reporting (reporting bias), and other potential sources of bias. Disagreements were resolved by discussion. For the quality of evidence assessment, two reviewers (M.T. and B.A.) adopted the Grading of Recommendations Assessment, Development, and Evaluation (GRADE) working group guidelines [34, 35]. Inconsistency, imprecision, indirectness, publication bias, and bias risk were considered. Our findings on the quality of evidence were justified, documented, and included in each outcome's reporting. Any disagreements were handled through consensus.

### Statistical analysis

The statistical analysis was carried out with RevMan v5.3 software [36]. We pooled dichotomous outcomes using risk ratio (RR) presented with the corresponding 95% confidence interval (CI) and continuous outcomes using mean difference (MD) with 95% CI. We used the I-square and Chi-square tests to examine heterogeneity; the Chi-square test determines if there is substantial heterogeneity, while the I-square determines the magnitude of heterogeneity. A substantial heterogeneity (for the Chi-square test) is defined as an alpha level below 0.1, according to the Cochrane Handbook (chapter nine) [30], while the I-square test is interpreted as follows: (0–40%: not significant; 30–60%: moderate heterogeneity; 50–90%: substantial heterogeneity; and 75–100%: considerable heterogeneity). We utilized the fixed-effects model. We also conducted a subgroup analysis depending on the time of assessment. Because we only included less than ten studies in each outcome, we did not conduct funnel plots to reveal publication bias, as advised by Egger et al. [37].

## Results

### Search results and study selection

We identified 1334 records after databases searching, then 430 duplicates were excluded. Title and abstract screening excluded 889 irrelevant records. We proceeded to full-text screening with 15 articles, 11 articles were excluded, and finally, only four articles met our inclusion criteria. The PRISMA flow chart of the detailed selection process is demonstrated in Figure S1.

### Characteristics of included studies

We included four trials [25–28] with a total of 339 participants who were randomized to either perioperative DEX (n = 170) or saline infusion (n = 169). Further included trials’ characteristics are presented in Table 1. DEX was administered after induction of anesthesia till the end of the operation in two trials [26, 27], with one trial administrating DEX for 15 min before the induction of anesthesia and until 30 min after it [25] and another until two hours after the end of surgery [28]. The mean age of the DEX group and the control group are (43.67 ± 22.57) and (43.33 ± 14.79), respectively. Female participants were a total of 142 (39.9%) divided between the DEX group and the control group, 70 (39.8%) and 72 (41.1%) participants, respectively. Further baseline characteristics of the participants are presented in Table 2.

**Table 1:** Summary characteristics of the included trials

Study ID	Study design	Country	Graft source	Total participants	DEX dose	Primary outcome	DGF definition	Rejection confirmation method
Liu et al. 2022 [25]	Single-center, double-blinded RCT	China	Deceased donors	65	Initial loading dose of (0.6 μg/kg IV) over 15 min before anesthesia, then infusion of (0.4 μg/kg/h) until 30 min after reperfusion of transplanted kidney	Concentrations of KIM-1, Cr, blood urea, β2-MG, CysC, eGFR), and urine output	Need for dialysis during the first week after transplantation	N/A
Park et al. 2021 [26]	Single-center, double-blinded RCT	South Korea	Living donors	103	Infusion of (0.4 μg/kg/h) starting immediately after anesthesia induction and until the end of surgery	Cr concentration on POD7	Need for dialysis during the first week after transplantation	Biopsy
Shan et al. 2022 [27]	Single-center, double-blinded RCT	China	Deceased donors	111	Infusion of (0.4 μg/kg/h) starting immediately after anesthesia induction and until the end of surgery	DGF	Need for dialysis during the first week after transplantation	Biopsy
Wang et al. 2022 [28]	Single-center, single-blinded RCT	Taiwan	Living donors^a^	60	Infusion of (0.1–0.7 μg/kg/h) according to the patient response, starting immediately after anesthesia induction and until two hours after the end of surgery	Cr concentration on POD2	N/A	N/A

*RCT* randomized controlled trials, *DEX* dexmedetomidine, *IV* intravenous, *DGF* delayed graft function, *KIM-1* urinary kidney injury molecule-1, *Cr* serum Creatinine, *β2-MG* β2 microglobulin, *CysC* Cystatin C, *eGFR* estimated glomerular filtration rate, *POD* postoperative day, *N/A* not available

^a^With 2 participants receiving kidney grafts from deceased donors

**Table 2:** Baseline characteristics of the participants

Study ID	No. of patients		Age (years) Mean (SD)		Gender (female) No. (%)		Comorbidity N. (%)				BMI Mean (SD)	
							HTN		DM			
	DEX	Control	DEX	Control	DEX	Control	DEX	Control	DEX	Control	DEX	Control
Liu et al. 2022 [25]	33	32	40.76 (8.78)	42.59 (9.49)	10 (30.30)	6 (18.75)	N/A	N/A	N/A	N/A	24.15 (3.87)	23.20 (3.07)
Park et al. 2021 [26]	51	52	50 (8.39)	48.33 (12.96)	28 (55)	29 (56)	45 (88)	45 (87)	18 (35)	14 (30)	N/A	N/A
Shan et al.2022 [27]	56	55	43.5 (10.7)	43.3 (10.9)	20 (35.7)	27 (49.1)	56 (100)	55 (100)	3 (5.4)	3 (5.5)	21.8 (3.2)	21.1 (3.2)
Wang et al. 2022 [28]	30	30	43.67 (22.57)	43.33 (14.79)	12 (40)	10 (33)	25 (83)	16 (53)	4 (13)	6 (20)	N/A	N/A

Study ID	Dialysis history						ABO incompatibility N. (%)		Creatinine Mg/dl Mean (SD)		Hear rate, bpm Mean (SD)	
	Duration Mean (SD)		Hemodialysis N. (%)		Peritoneal dialysis N. (%)							
	DEX	Control	DEX	Control	DEX	Control	DEX	Control	DEX	Control	DEX	Control
Liu et al. 2022 [25]	N/A	N/A	N/A	N/A	N/A	N/A	N/A	N/A	11.24 (3.74)	10.64 (3.22)	79.97 (14)	77.94 (13.7)
Park et al. 2021 [26]	2.33 (3.81)	2.33 (3.81)	41 (80)	40 (77)	6 (12)	6 (12)	24 (47)	19 (37)	5.9 (1.98)	6.13 (1.83)	62 (7.63)	66 (13.72)
Shan et al.2022 [27]	23.2 (20.16)	31 (34.26)	34 (61)	38 (69)	22 (39)	17 (31)	N/A	N/A	10.52 (3.00)	10.28 (3.41)	82.3 (13.1)	80.5 (12.9)
Wang et al. 2022 [28]	N/A	N/A	19 (63)	17 (57)	11 (37)	13 (43)	8 (27)	9 (30)	11.3 (4.1)	11.5 (4.5)	86 (14.79)	87 (20.24)

*DEX* dexmedetomidine, *N/A* not available, *N* number, *SD* standard deviation, *bpm* beat per minute

### Risk of bias and quality of evidence

We assessed the quality of the included studies according to the Cochrane risk of bias tool, as shown in Figure S2. All of the included trials had a low risk of selection bias except Liu et al. [25], with a high risk of selection bias. Moreover, all included trials had a low risk of performance and detection biases except Wang et al. [28], with a high risk of performance and detection biases. Also, all of the included trials had a low risk of attrition bias except Liu et al. [25], with a high risk of attrition bias. Furthermore, all included trials had a low risk of reporting bias except Liu et al. [25], with an unclear risk of reporting bias. Finally, all of the included trials had a low risk of other biases. Author judgments are furtherly clarified in Table S3.

Using the GRADE system, all the included primary outcomes yielded low-quality evidence. Details and explanations are clarified in Table S4.

### Primary outcomes

#### DGF

**Fig. 1:**
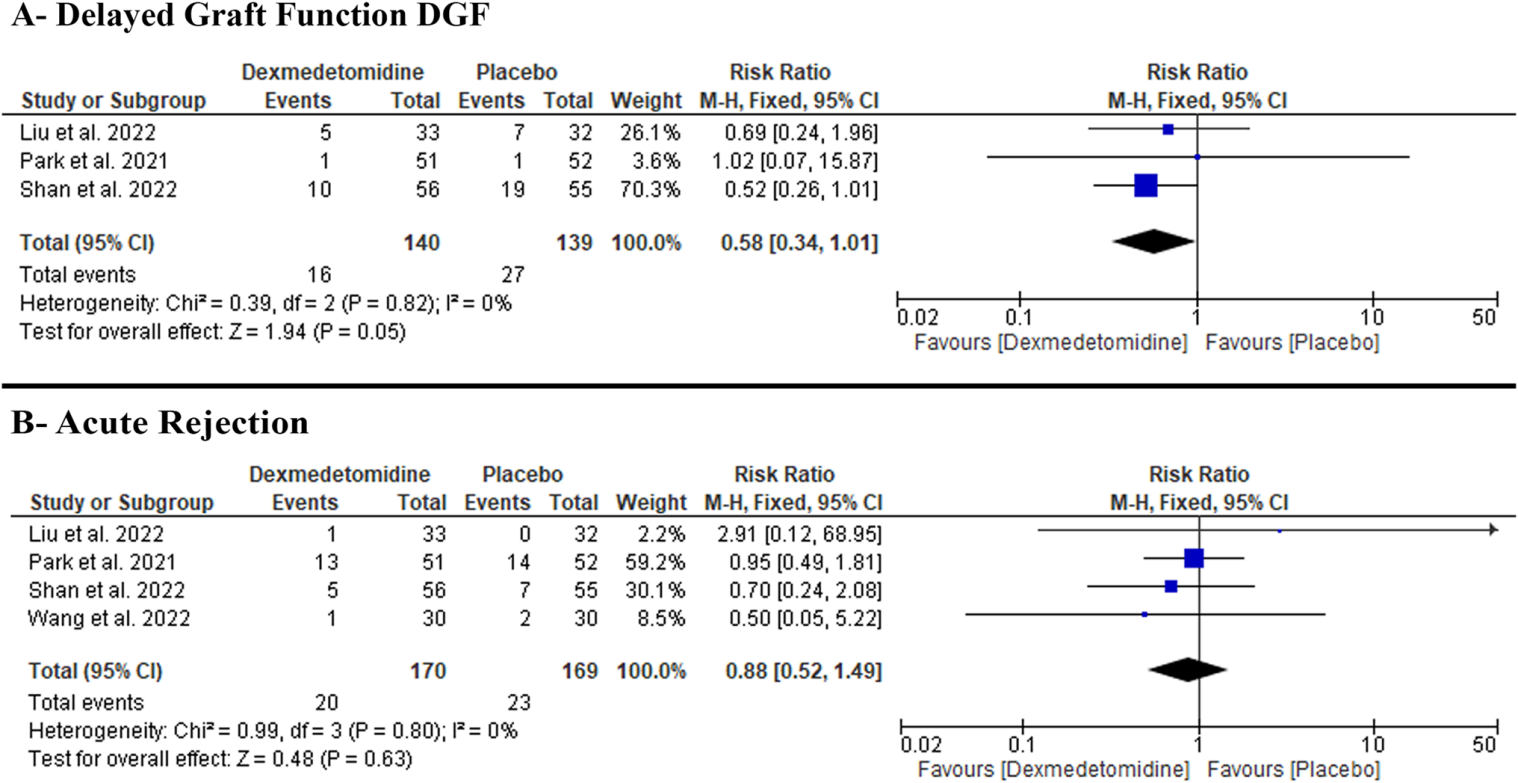
Forest plot of the primary outcomes [**A**: delayed graft function (DGF), **B**: acute rejection]. *I2* I-squared, *CI* confidence interval

We found no difference between DEX and placebo regarding the incidence of DGF (RR: 0.58 with 95% CI [0.34, 1.01], p = 0.05) (low-quality evidence) (Fig. 1A, Table S4). The pooled studies were homogenous (p = 0.81, I-square = 0%).

#### Acute rejection

We found no difference between DEX and placebo regarding the incidence of acute rejection (RR: 0.88 with 95% CI [0.52, 1.49], p = 0.63) (low-quality evidence) (Fig. 1B, Table S4). The pooled studies were homogenous (p = 0.8, I-square = 0%).

### Secondary outcomes

#### Creatinine (mg/dl)

**Fig. 2:**
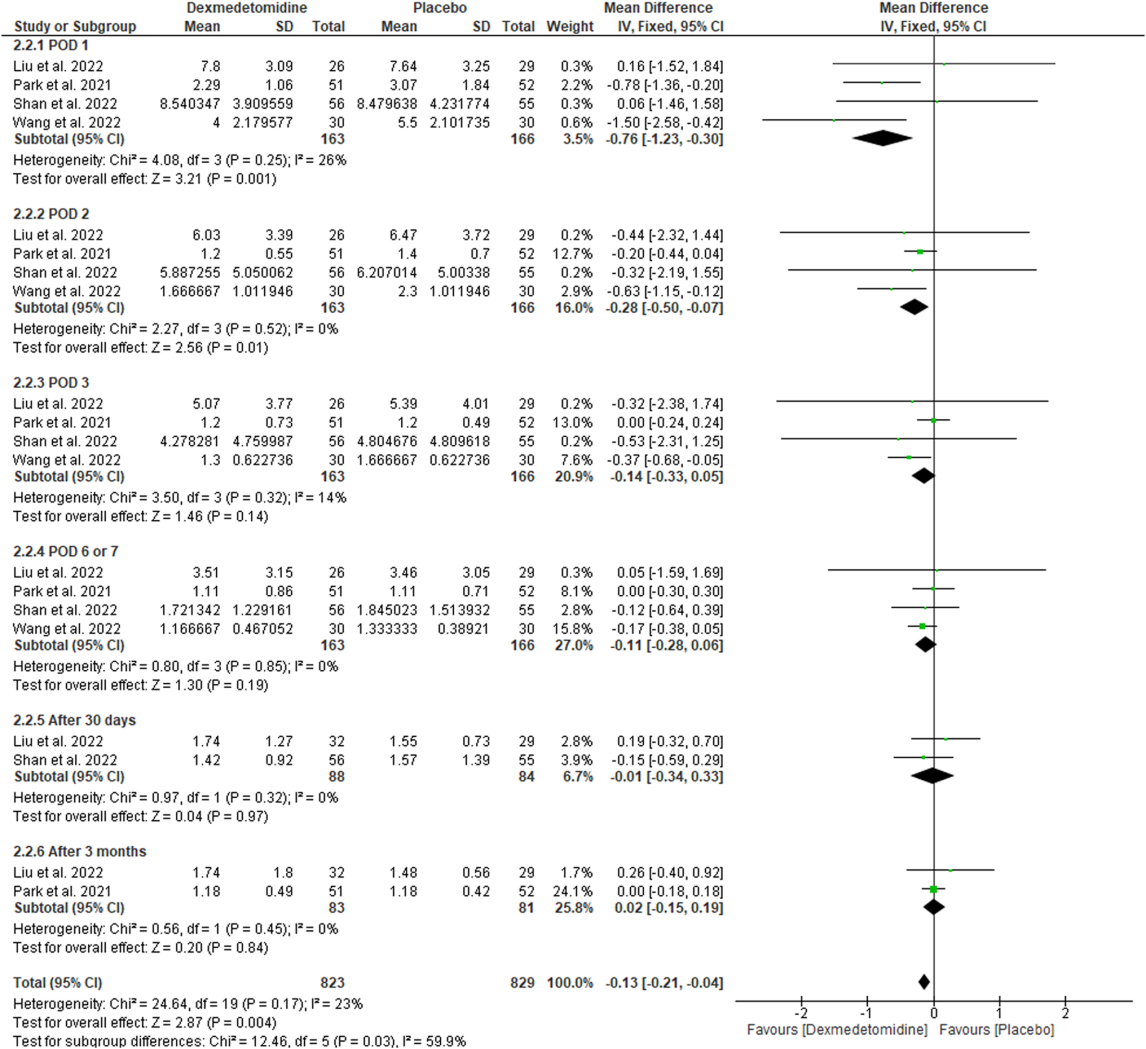
Forest plot of creatinine (mg/dl). *I2* I-squared, *CI* confidence interval

The pooled mean difference favored DEX over placebo on POD 1 (MD: − 0.76 with 95% CI [− 1.23, − 0.3], p = 0.001), POD 2 (MD: − 0.28 with 95% CI [− 0.5, − 0.07], p = 0.01); however, we found no difference between DEX and placebo on POD 3 (MD: − 0.14 with 95% CI [− 0.33, 0.05], p = 0.14), POD 6/7 (MD: − 0.11 with 95% CI [− 0.28, 0.06], p = 0.19), after 30 days (MD: − 0.01 with 95% CI [− 0.34, 0.33], p = 0.97), and after three months (MD: 0.02 with 95% CI [− 0.15, 0.19], p = 0.84) (Fig. 2). Our results were homogenous with (p > 0.1, I-square > 50%).

#### Urine output (mL/h)

**Fig. 3:**
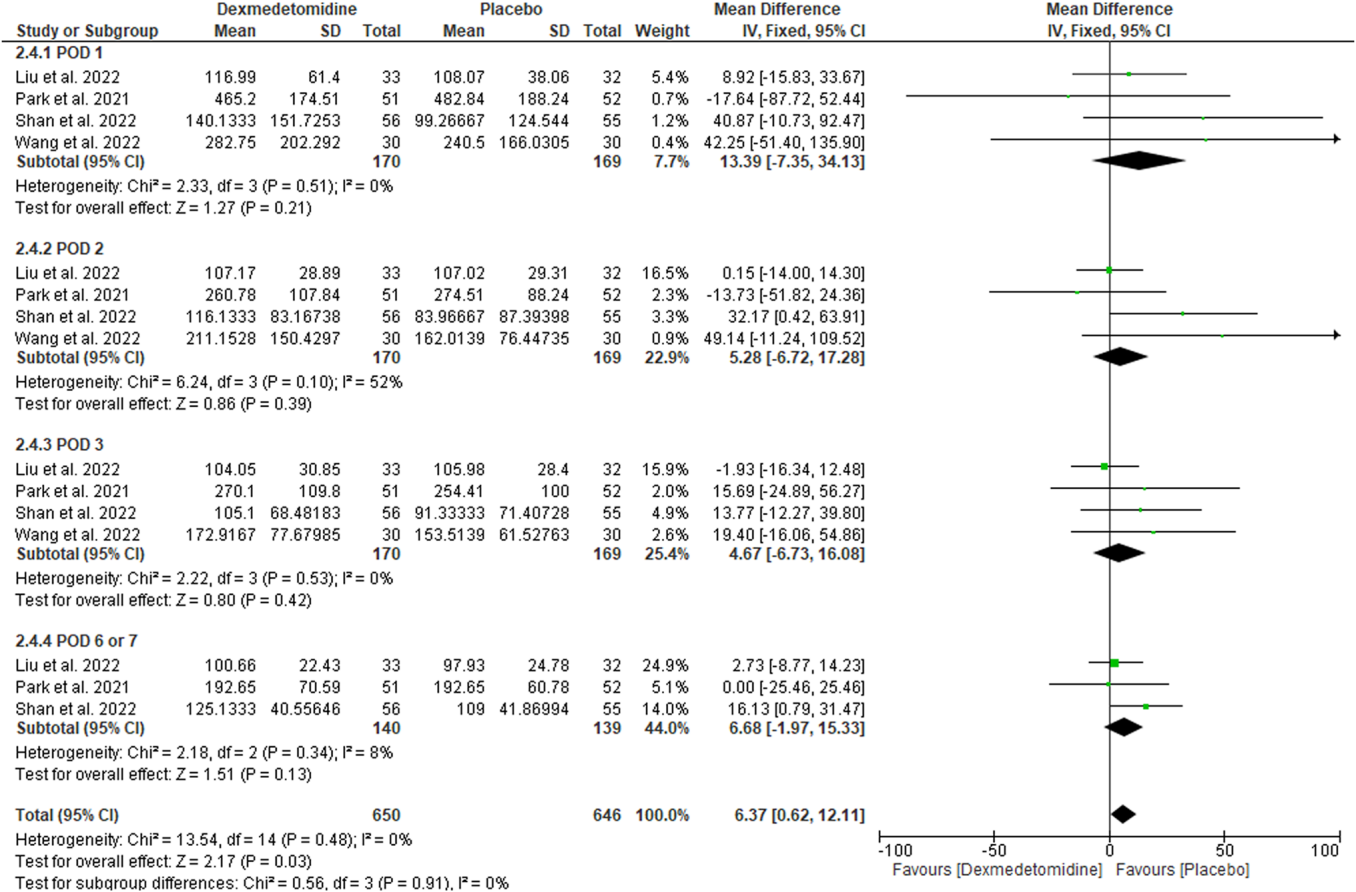
Forest plot of urine output (mL/h). *I2* I-squared, *CI* confidence interval

We found no difference between DEX and placebo on POD 1 (MD: 13.39 with 95% CI [− 7.35, 34.13], p = 0.21), POD 2 (MD: 5.28 with 95% CI [− 6.72, 17.28], p = 0.39), POD 3 (MD: 4.67 with 95% CI [− 6.73, 16.08], p = 0.42), and POD 6/7 (MD: 6.68 with 95% CI [− 1.97, 15.33], p = 0.13) (Fig. 3). Our results were homogenous with (p > 0.1, I-square > 50%).

#### Cystatin C (mg/L)

We found no difference between DEX and placebo on POD 1 (MD: − 0.15 with 95% CI [− 0.34, 0.05], p = 0.15), POD 2 (MD: − 0.02 with 95% CI [− 0.19, 0.16], p = 0.86), and after 30 days (MD: − 0.09 with 95% CI [− 0.29, 0.11], p = 0.38) (Figure S3). Our results were homogenous with (p > 0.1, I-square = 0%).

#### BUN (mg/dl)

The pooled mean difference favored DEX over placebo on POD 2 (MD: − 10.16 with 95% CI [− 17.21, − 3.10], p = 0.005), POD 3 (MD: − 6.72 with 95% CI [− 12.85, − 0.58], p = 0.03); however, we found no difference between DEX and placebo on POD 1 (MD: − 8.40 with 95% CI [− 17.98, 1.18], p = 0.09), POD 6/7 (MD: − 1.09 with 95% CI [− 6.80, 4.63], p = 0.71) (Figure S4). Our results were homogenous with (p > 0.1, I-square > 50%).

#### eGFR (mL/min/1.73 m^2^)

We found no difference between DEX and placebo on POD 6/7 (MD: − 4.09 with 95% CI [− 12.41, 4.24], p = 0.34), after 30 days (MD: − 0.14 with 95% CI [− 7.27, 7.00], p = 0.97), and after three months (MD: 0.30 with 95% CI [− 5.47, 6.06, p = 0.92) (Figure S5). Our results were homogenous with (p > 0.1, I-square = 0%).

## Discussion

DEX has been reported to have reno-protective effects in different literature [16–22]. After analyzing the pooled data from the included RCTs [25–28], we found that perioperative infusion of DEX in patients undergoing renal transplantation decreased serum creatinine levels in POD1 and POD2 and serum BUN levels in POD2 and POD3, compared to placebo. However, we found no superiority of DEX compared to placebo in decreasing the incidences of DGF and acute graft rejection. Furthermore, postoperative levels of eGFR; cystatin c; urine output; BUN in POD 1 and POD 6/7; and creatinine in POD3, POD 6/7, POD 30, and POD 90 were similar in both DEX and placebo groups.

The reno-protective effects of DEX have been extensively investigated with multiple proposed mechanisms. First, DEX can prevent adrenergic vasoconstriction responses in the kidney and promote nitric oxide-dependent vasodilatation, sustaining glomerular filtration and renal blood flow [25, 38, 39]. Second, DEX inhibits ERK1/2 and NF-κB and modulates inflammatory cytokines decreasing TNF and IL-6, hence attenuating the systematic inflammatory response [25, 40–42]. Third, Liu et al. [25] detected decreased levels of kidney injury molecule 1 (KIM-1) with DEX compared to placebo. To clarify, KIM-1 is considered an ideal biomarker of kidney injury [25]. Moreover, KIM-1 has been reported to be a reliable predictor of inflammatory kidney injury and hence long-term graft survival [43, 44]. Finally, DEX can promote autophagy by reducing NLRP3 inflammasome activation; therefore, DEX ameliorates kidney IRI after transplantation [14].

Despite the previous reno-protective effects of DEX, our pooled analysis found no difference in DGF incidence. We can attribute this difference to the fact that most clinical trials evaluating the reno-protective effects of DEX excluded patients with deteriorated renal functions [20, 45, 46]. In this regard, assessing the reno-protective impact of DEX during kidney transplantation surgery in patients with renal impairment experiencing cold kidney ischemia may show different findings [25]. However, in a retrospective cohort, Chen et al. [29] reported decreased incidence of post-operative DGF with perioperative DEX injection. DGF in renal transplantation results from IRI and activation of the immune system. DGF is associated with biopsy-proven acute graft rejection [8], increased graft immunogenicity, decreased graft survival, and chronic graft failure [47]. Accordingly, preventing DGF in the early phase after renal transplantation is very critical in determining the long-term prognosis. To date, there is no FDA-approved treatment for DGF prevention, up to our knowledge. However, given our understanding of the DGF pathophysiology, measures decreasing the IRI, including vasodilators, antioxidants, anti-inflammatory, and immunosuppressive therapies, might help decrease its incidence.

Besides DGF, acute rejection is another complication that occurs because of IRI [7, 8]. Recipients with DGF are at a higher risk of developing acute graft rejection, with a 49% incidence of acute rejection in patients with DGF compared to 35% in those without DGF [48]. In line with our DGF findings, DEX was not different from the placebo in preventing acute graft rejection; however, Chen et al. [22] reported that perioperative DEX decreased the incidence of acute rejection in the early port transplantation phase. The rationale behind this difference is unclear, but our pooled analysis included few patients compared to Chen et al. [22]; hence, our analysis can be underpowered to detect this effect. Another reasonable rationale is that the ischemic injury of the graft starts directly after kidney organ recovery [49]. Accordingly, pre-treatment with DEX in the recipient alone is probably inadequate to prevent IRI, which is a pervasive limitation in the clinical research of organ transplantation [26].

Creatinine (Cr) level is also associated with the allograft function [50, 51]. It is suggested that a 0.3 mg/dl (25 μmol/l) increase in the serum Cr from baseline is an indicator of acute kidney injury and is associated with increased mortality risk and other adverse outcomes [52, 53]. Similarly, Pascual et al. [54] reported that early change in the serum Cr after transplantation was strongly correlated with long-term graft survival (> 10 years). To detect the early changes in the graft’s function, Park et al. [26] also targeted a 0.3 mg/dl (25 μmol/l) change in serum Cr levels. Compared to the placebo, DEX was superior in the short-term in the first 48 h after transplantation; however, DEX did not show superior effects in the post-transplantation serum Cr in POD7, after three months, and after six months.

Moreover, higher urine output early post-transplantation is associated with favorable graft outcomes [55]. It is difficult to determine the baseline urine output early after transplantation; however, it tends to stable by the first month [55]. Given the alpha-2-adrenoreceptor agonist activity of DEX, it can inhibit renin secretion and increase urine output. The use of DEX was associated with increased urine output in the first 24 h after coronary artery bypass graft surgery [55]. However, in our analysis, we did not find a significant increase in urine output in the DEX group compared to the placebo after transplantation. Of the included studies, only Shan et al. [27] reported an increase in urine output in the DEX group in POD2 and POD7.

Cystatin c inhibits lysosomal cysteine proteinases, and multiple studies have suggested its superiority in calculating GFR to determine renal function [56]. In a study conducted by White et al., GFR measurements derived from cystatin C were demonstrated to be more accurate than creatinine-based GFR measurements in kidney transplant patients when compared to the measured GFR [57]. Similarly, current eGFR equations which rely on creatinine measurements have been shown to lack accuracy in kidney transplant patients [32]. However, in our study, no difference in Cystatin C was observed between DEX treatment and placebo groups in POD 1, POD 2, or after 30 days. In the same line, a meta-analysis conducted by Shlikpak et al. involving 11 general-population studies and five studies of chronic kidney disease cohorts showed that utilizing cystatin c independently or in addition to creatinine when calculating eGFR ameliorates the usage of eGFR to assess the risk of ESRD as well as death [58]. Hence, we believe that further studying the utilization of cystatin c to evaluate kidney function in transplant patients may lead to a more accurate assessment of kidney graft function and improvements in patient outcomes.

BUN is a marker that is associated with urea excretion the excretory functions of the kidney [58]. Notably, Seki et al. conducted a study on patients with chronic kidney disease and found that increased BUN levels were associated with negative kidney outcomes irrespective of eGFR values [59]. Their findings suggested that BUN levels may play a greater role in evaluating renal functions in patients with chronic kidney disease than previously considered [59]. In our study, we found that the pooled mean difference of BUN favored the DEX as compared to the placebo on POD 2 and 3. However, no significant difference was exhibited between the DEX and placebo groups on POD 1. Although independent BUN levels cannot necessarily be indicative of renal function, the benefits observed on POD 2 and 3 with DEX treatment may be suggestive of improved excretory kidney function post-graft transplantation. Combined with the evidence from Seki et al. [59], we believe that BUN levels may be accurately considered in the kidney transplantation patient population and aid in the accurate assessment of renal function.

GFR is conventionally used to assess renal function as well as identify kidney disease stages. Many different equations have been determined to calculate GFR, which largely rely on creatinine levels, which can be influenced by several factors such as hydration, metabolic function, and drug interactions [60]. Estimated GFR or eGFR calculated using both creatinine and cystatin c levels have demonstrated greater accuracy as opposed to using one or the other [58, 60]. Moreover, creatinine-based eGFR is less accurate in patients with lower GFR or chronic kidney disease, and thus utilizing both cystatin c, and creatinine has been strongly suggested to accurately determine renal function in these patients [58, 60]. In our study, the results demonstrated no difference in eGFR between DEX or placebo groups in any of the time intervals, including POD 6, POD 7, after 30 days, or after three months. Based solely on these results, DEX treatment did not seem to influence renal function. As referenced earlier, eGFR loses its accuracy in patients with chronic kidney disease and determined renal function, so our results regarding eGFR values may not be indicative of true renal function, especially as subjects are post-graft transplantation.

In comparison with other procedures, perioperative infusion of DEX showed different results. Some studies reported a decrease in the incidence of acute kidney injury [20, 45, 46], while others reported no renal benefits of its use [55, 61, 62]. Perioperative DEX in liver transplantation decreased the IRI and improved graft outcomes through its sedative and immunosuppressive effects [63].

Despite the protective effects of DEX on IRI, DEX perioperative use is usually associated with the incidence of clinically significant bradycardia and hypotension [23, 24], and low cardiac output or low blood pressure can impair microcirculation [64]. Shan et al. [27] is the only included RCT that reported the incidence of bradycardia (16.1% vs. 9.1%) and hypotension (14.3% vs. 10.9%) in DEX and placebo groups, respectively, without statistically significant association [27]. Moreover, Wang et al. found that DEX has not affected the patency of sublingual microcirculation, implying the safety of DEX during kidney transplantation [28]. However, Liu et al. reported that DEX was associated with bradycardia without reporting clear data, and all cases were successfully treated with atropine [25].

## Strengths

Our meta-analysis is the first to address the reno-protective effects of perioperative DEX in renal transplantation, according to our best knowledge. We also adhered to PRISMA guidelines while conducting this review [29]. Furthermore, we conducted a quality of evidence assessment using the most recent GRADE guidelines [34, 35].

## Limitations

Our study has a few limitations: first, we only included four single-center RCTs with a relatively small number of participants. Second, several factors might alter the effect of DEX on kidney transplantation outcomes, including drug interactions, living versus deceased donors, post-transplantation complications, and management [63]. Third, DEX dosage and duration of perioperative infusion varied across the included RCTs, which may confound our findings. Fourth, we could not add the outcomes of bradycardia and hypotension in our meta-analysis as only one RCT [27] reported them. Finally, DGF assessment is dependent on physicians’ subjective experience, which may affect their decision on whether to dialyze graft recipients or not [26].

## Conclusion

Evidence of DEX’s reno-protective effects in kidney transplantation is uncertain, with no difference compared to placebo in preventing DGF and acute rejection. However, we found statistically significant improvement in the short-term serum creatinine and blood urea nitrogen which warrants more multi-center, large-scale clinical trials to furtherly investigate the reno-protective effects of DEX, especially in the long-term.

## Supplementary Information

Below is the link to the electronic supplementary material.

Supplementary file1 (DOCX 1322 KB)

## Data Availability

The datasets used and/or analysed during the current study are available from the corresponding author on reasonable request.
