# Supplementary material for: Reno-protective effects of perioperative dexmedetomidine in kidney transplantation: a systematic review and meta-analysis of randomized controlled trials
Source: Int Urol Nephrol. 2023 Mar 30;55(10):2545–56. doi: 10.1007/s11255-023-03568-3 (PMC10499682; doi:10.1007/s11255-023-03568-3)
Supplement: Supplementary file 1 — Supplementary file1 (DOCX 1322 KB) [file 11255_2023_3568_MOESM1_ESM.docx]

**Supplementary material:**

**Title.**

**Reno-Protective Effects of Perioperative Dexmedetomidine in Kidney Transplantation: A Systematic Review and Meta-Analysis of Randomized Controlled Trials.**

**Running Title.**

Dexmedetomidine in Kidney Transplantation.

**Authors.**Mohamed T. Abuelazm^1^, Ahmed Ghanem^2^, Amit Johanis^3^, Abdelrahman Mahmoud^4^, Abdul Rhman Hassan^1^, Basant E. Katamesh^1^, Mostafa Atef^5^, Basel Abdelazeem^6,7^.

**Affiliations.**

1. Faculty of Medicine, Tanta University, Tanta, Egypt.

2. Cardiology Department, The Lundquist Institute, Torrance, CA, USA.

3. Faculty of Medicine, Creighton University, Phoenix, AZ, USA.

4. Faculty of Medicine, Minia University, Minia, Egypt.

5. Faculty of Medicine, Cairo University, Cairo, Egypt.

6. Department of Internal Medicine, McLaren Health Care, Flint, Michigan, USA.

7. Department of Internal Medicine, Michigan State University, East Lansing, Michigan, USA.

**Keywords.**

DEX; Dexmedetomidine; Kidney Transplantation; Systematic Review; Meta-Analysis.

**Contents:**

**Tables.**

Table S1: PRISMA 2020 checklist.

Table S2: Search terms and results in different databases.

Table S3: Author judgments for ROB assessment.

Table S4: GRADE evidence profile of the primary outcomes.

**Figures.**

Figure S1: PRISMA flow chart of the screening process.

Figure S2: Summary of risk of bias (A- review authors' judgments about each risk of bias item for each included study, B- review authors' judgments about each risk of bias item presented as percentages across all included studies).

Figure S3: Forest plot of comparison: 2 Secondary Outcomes, outcome: 2.3 Cystatin C (mg/L).

Figure S4: Forest plot of comparison: 2 Secondary Outcomes, outcome: 2.5 BUN (mg/dl).

Figure S5: Forest plot of comparison: 2 Secondary Outcomes, outcome: 2.6 eGFR (mL/min/1.73 m2).

| **Section and Topic** | **Item #** | **Checklist item** | **Location where item is reported** |
| --- | --- | --- | --- |
| **TITLE** | | |  |
| Title | 1 | Identify the report as a systematic review. | Line 2 |
| **ABSTRACT** | | |  |
| Abstract | 2 | See the PRISMA 2020 for Abstracts checklist. | Page 3 |
| **INTRODUCTION** | | |  |
| Rationale | 3 | Describe the rationale for the review in the context of existing knowledge. | Page 5 |
| Objectives | 4 | Provide an explicit statement of the objective(s) or question(s) the review addresses. | Page 6 |
| **METHODS** | | |  |
| Eligibility criteria | 5 | Specify the inclusion and exclusion criteria for the review and how studies were grouped for the syntheses. | Page 7, subsection 2.3 |
| Information sources | 6 | Specify all databases, registers, websites, organisations, reference lists and other sources searched or consulted to identify studies. Specify the date when each source was last searched or consulted. | Page 7, subsection 2.2 |
| Search strategy | 7 | Present the full search strategies for all databases, registers and websites, including any filters and limits used. | Supplementary material, table S2 |
| Selection process | 8 | Specify the methods used to decide whether a study met the inclusion criteria of the review, including how many reviewers screened each record and each report retrieved, whether they worked independently, and if applicable, details of automation tools used in the process. | Page 8, subsection 2.4 |
| Data collection process | 9 | Specify the methods used to collect data from reports, including how many reviewers collected data from each report, whether they worked independently, any processes for obtaining or confirming data from study investigators, and if applicable, details of automation tools used in the process. | Page 8, subsection 2.5 |
| Data items | 10a | List and define all outcomes for which data were sought. Specify whether all results that were compatible with each outcome domain in each study were sought (e.g. for all measures, time points, analyses), and if not, the methods used to decide which results to collect. | Page 8, subsection 2.5 |
|  | 10b | List and define all other variables for which data were sought (e.g. participant and intervention characteristics, funding sources). Describe any assumptions made about any missing or unclear information. | Page 8, subsection 2.5 |
| Study risk of bias assessment | 11 | Specify the methods used to assess risk of bias in the included studies, including details of the tool(s) used, how many reviewers assessed each study and whether they worked independently, and if applicable, details of automation tools used in the process. | Page 8, subsection 2.6 |
| Effect measures | 12 | Specify for each outcome the effect measure(s) (e.g. risk ratio, mean difference) used in the synthesis or presentation of results. | Page 9, subsection 2.7 |
| Synthesis methods | 13a | Describe the processes used to decide which studies were eligible for each synthesis (e.g. tabulating the study intervention characteristics and comparing against the planned groups for each synthesis (item #5)). | Page 9, subsection 2.7 |
|  | 13b | Describe any methods required to prepare the data for presentation or synthesis, such as handling of missing summary statistics, or data conversions. | Page 9, subsection 2.7 |
|  | 13c | Describe any methods used to tabulate or visually display results of individual studies and syntheses. | Page 9, subsection 2.7 |
|  | 13d | Describe any methods used to synthesize results and provide a rationale for the choice(s). If meta-analysis was performed, describe the model(s), method(s) to identify the presence and extent of statistical heterogeneity, and software package(s) used. | Page 9, subsection 2.7 |
|  | 13e | Describe any methods used to explore possible causes of heterogeneity among study results (e.g. subgroup analysis, meta-regression). | Page 9, subsection 2.7 |
|  | 13f | Describe any sensitivity analyses conducted to assess robustness of the synthesized results. | Page 9, subsection 2.7 |
| Reporting bias assessment | 14 | Describe any methods used to assess risk of bias due to missing results in a synthesis (arising from reporting biases). | Page 8, subsection 2.6 |
| Certainty assessment | 15 | Describe any methods used to assess certainty (or confidence) in the body of evidence for an outcome. | Page 8, subsection 2.6 |
| **RESULTS** | | |  |
| Study selection | 16a | Describe the results of the search and selection process, from the number of records identified in the search to the number of studies included in the review, ideally using a flow diagram. | Page 10, subsection 3.1 |
|  | 16b | Cite studies that might appear to meet the inclusion criteria, but which were excluded, and explain why they were excluded. | Not applicable |
| Study characteristics | 17 | Cite each included study and present its characteristics. | Page 10, subsection 3.2 |
| Risk of bias in studies | 18 | Present assessments of risk of bias for each included study. | Page 10, subsection 3.3 |
| Results of individual studies | 19 | For all outcomes, present, for each study: (a) summary statistics for each group (where appropriate) and (b) an effect estimate and its precision (e.g. confidence/credible interval), ideally using structured tables or plots. | Pages 11-12, subsections 3.4-3.5 |
| Results of syntheses | 20a | For each synthesis, briefly summarise the characteristics and risk of bias among contributing studies. | Page 10, subsection 3.3 |
|  | 20b | Present results of all statistical syntheses conducted. If meta-analysis was done, present for each the summary estimate and its precision (e.g. confidence/credible interval) and measures of statistical heterogeneity. If comparing groups, describe the direction of the effect. | Pages 11-12, subsections 3.4-3.5 |
|  | 20c | Present results of all investigations of possible causes of heterogeneity among study results. | Pages 11-12, subsections 3.4-3.5 |
|  | 20d | Present results of all sensitivity analyses conducted to assess the robustness of the synthesized results. | Not applicable |
| Reporting biases | 21 | Present assessments of risk of bias due to missing results (arising from reporting biases) for each synthesis assessed. | Page 10, subsection 3.3 |
| Certainty of evidence | 22 | Present assessments of certainty (or confidence) in the body of evidence for each outcome assessed. | Table 3 |
| **DISCUSSION** | | |  |
| Discussion | 23a | Provide a general interpretation of the results in the context of other evidence. | Page 13 |
|  | 23b | Discuss any limitations of the evidence included in the review. | Page 18 |
|  | 23c | Discuss any limitations of the review processes used. | Page 18 |
|  | 23d | Discuss implications of the results for practice, policy, and future research. | Page 18 |
| **OTHER INFORMATION** | | |  |
| Registration and protocol | 24a | Provide registration information for the review, including register name and registration number, or state that the review was not registered. | Page 7, subsection 2.1 |
|  | 24b | Indicate where the review protocol can be accessed, or state that a protocol was not prepared. | Page 7, subsection 2.1 |
|  | 24c | Describe and explain any amendments to information provided at registration or in the protocol. | Page 7, subsection 2.1 |
| Support | 25 | Describe sources of financial or non-financial support for the review, and the role of the funders or sponsors in the review. | Page 19 |
| Competing interests | 26 | Declare any competing interests of review authors. | Page 18 |
| Availability of data, code and other materials | 27 | Report which of the following are publicly available and where they can be found: template data collection forms; data extracted from included studies; data used for all analyses; analytic code; any other materials used in the review. | Page 19 |

Table S1: PRISMA 2020 checklist.

| Database | Search Terms | Search Field | Search Results |
| --- | --- | --- | --- |
| PubMed | (dexmedetomidine OR DEX OR precedes OR MPV-1440 OR "MPV 1440" OR MPV1440 OR "Dexmedetomidine Hydrochloride") AND ("Renal Transplantation*" OR "Kidney Grafting" OR "Kidney Transplantation*") | All Field | 654 |
| Cochrane | (dexmedetomidine OR DEX OR precedes OR MPV-1440 OR "MPV 1440" OR MPV1440 OR "Dexmedetomidine Hydrochloride") AND ("Renal Transplantation*" OR "Kidney Grafting" OR "Kidney Transplantation*") | All Field | 73 |
| WOS | (dexmedetomidine OR DEX OR precedes OR MPV-1440 OR "MPV 1440" OR MPV1440 OR "Dexmedetomidine Hydrochloride") AND ("Renal Transplantation*" OR "Kidney Grafting" OR "Kidney Transplantation*") | All Field | 423 |
| SCOPUS | TITLE-ABS-KEY ((dexmedetomidine  OR  dex  OR  precedes  OR  mpv-1440  OR  "MPV 1440"  OR  mpv1440  OR  "Dexmedetomidine Hydrochloride" )  AND  ( "Renal Transplantation*"  OR  "Kidney Grafting"  OR  "Kidney Transplantation*" )) | Title, Abstract, Keywords | 104 |
| EMBASE | #3.  #1 AND #2  #2.  'renal transplantation':ti,ab,kw OR 'kidney       grafting':ti,ab,kw OR 'kidney       transplantation':ti,ab,kw  #1.  dexmedetomidine:ti,ab,kw OR dex:ti,ab,kw OR       precedes:ti,ab,kw OR 'mpv 1440':ti,ab,kw OR       mpv1440:ti,ab,kw OR 'dexmedetomidine       hydrochloride':ti,ab,kw  ....................................................... | All Field | 80 |

Table S2: Search terms and results in different databases.

| **Study ID** | **Domain** | **Judgment** |
| --- | --- | --- |
| Liu et al. 2022 | Random sequence generation (selection bias) | High risk “they tossed a coin to decide who would use DEX according to the hospital ID of the patients” |
|  | Allocation concealment (selection bias) | High risk “they tossed a coin to decide who would use DEX according to the hospital ID of the patients” |
|  | Incomplete outcome data (attrition bias) | High risk “unbalanced loss of follow-up “ |
|  | Selective reporting (reporting bias) | Unclear risk “no protocol” |
| Wang et al 2022 | Blinding of participants and personnel (performance bias) | High risk “single-blinded” |
|  | Blinding of outcome assessment (detection bias) | High risk “the assessors were not blinded, and the outcome can be affected by this, this was based on that the study is open-label” |

Table S3: Author judgments for ROB assessment.

| **Certainty assessment** | | | | | | | **№ of patients** | | **Effect** | | **Certainty** | **Importance** |
| --- | --- | --- | --- | --- | --- | --- | --- | --- | --- | --- | --- | --- |
| **№ of studies** | **Study design** | **Risk of bias** | **Inconsistency** | **Indirectness** | **Imprecision** | **Other considerations** | **[intervention]** | **[comparison]** | **Relative (95% CI)** | **Absolute (95% CI)** |  |  |
| **Delayed Graft Function** | | | | | | | | | | | | |
| 3 | randomised trials | not serious | not serious | not serious | Very serious^a^ | none | 16/146 (11.0%) | 27/146 (18.5%) | **RR 0.59** (0.34 to 1.02) | **76 fewer per 1,000** (from 122 fewer to 4 more) | ⨁⨁◯◯ Low | Critical |
| **Acute Rejection** | | | | | | | | | | | | |
| 4 | randomised trials | not serious | not serious | not serious | Very serious^a^ | none | 20/176 (11.4%) | 23/176 (13.1%) | **RR 0.88** (0.52 to 1.50) | **16 fewer per 1,000** (from 63 fewer to 65 more) | ⨁⨁◯◯ Low | Critical |

*Table S4: GRADE evidence profile of the primary outcomes.*

CI: confidence interval; RR: risk ratio

Explanations

a. The analysis included few events; less than 400 participants.


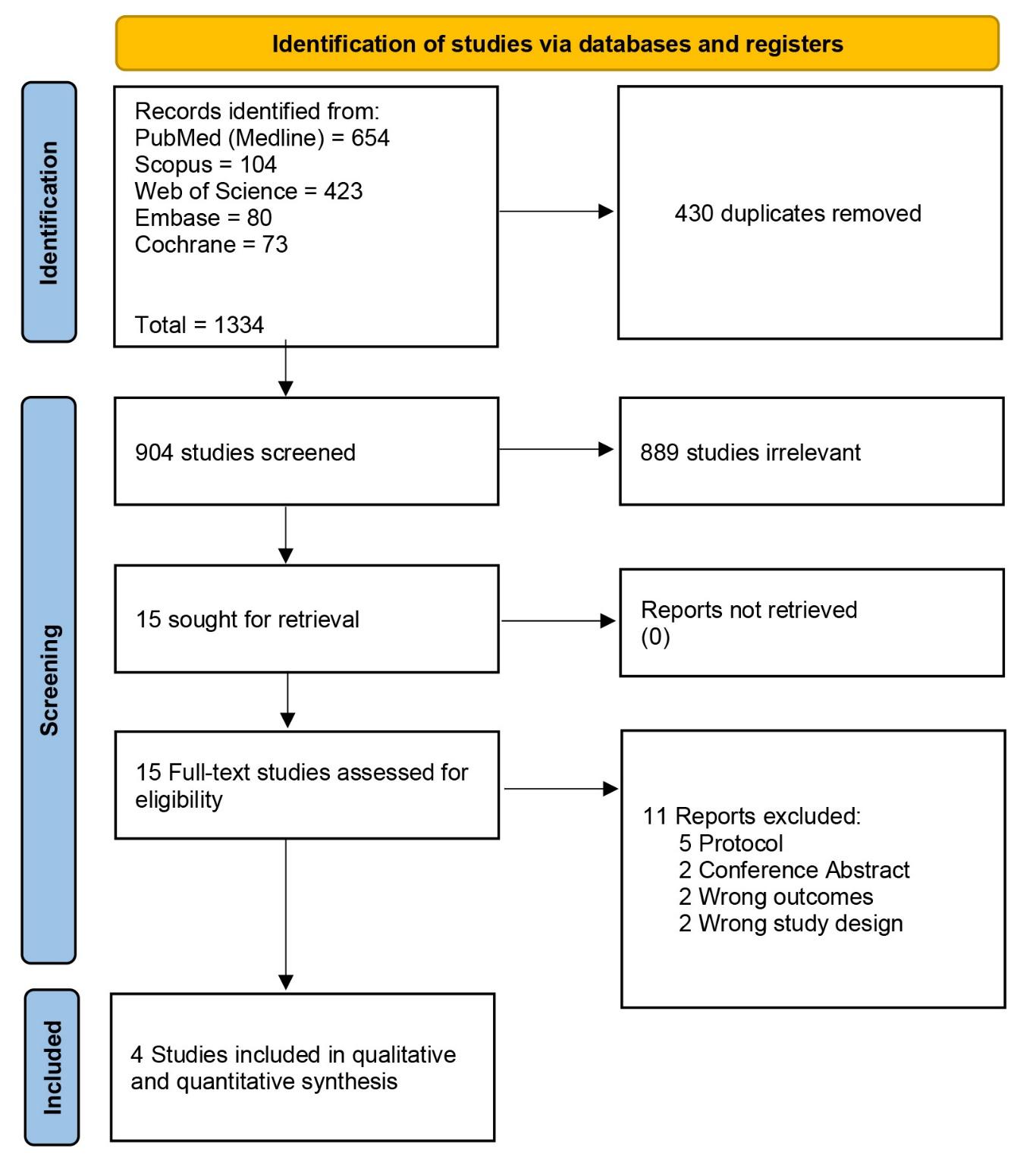


*Figure S1: PRISMA flow chart of the screening process.*

**
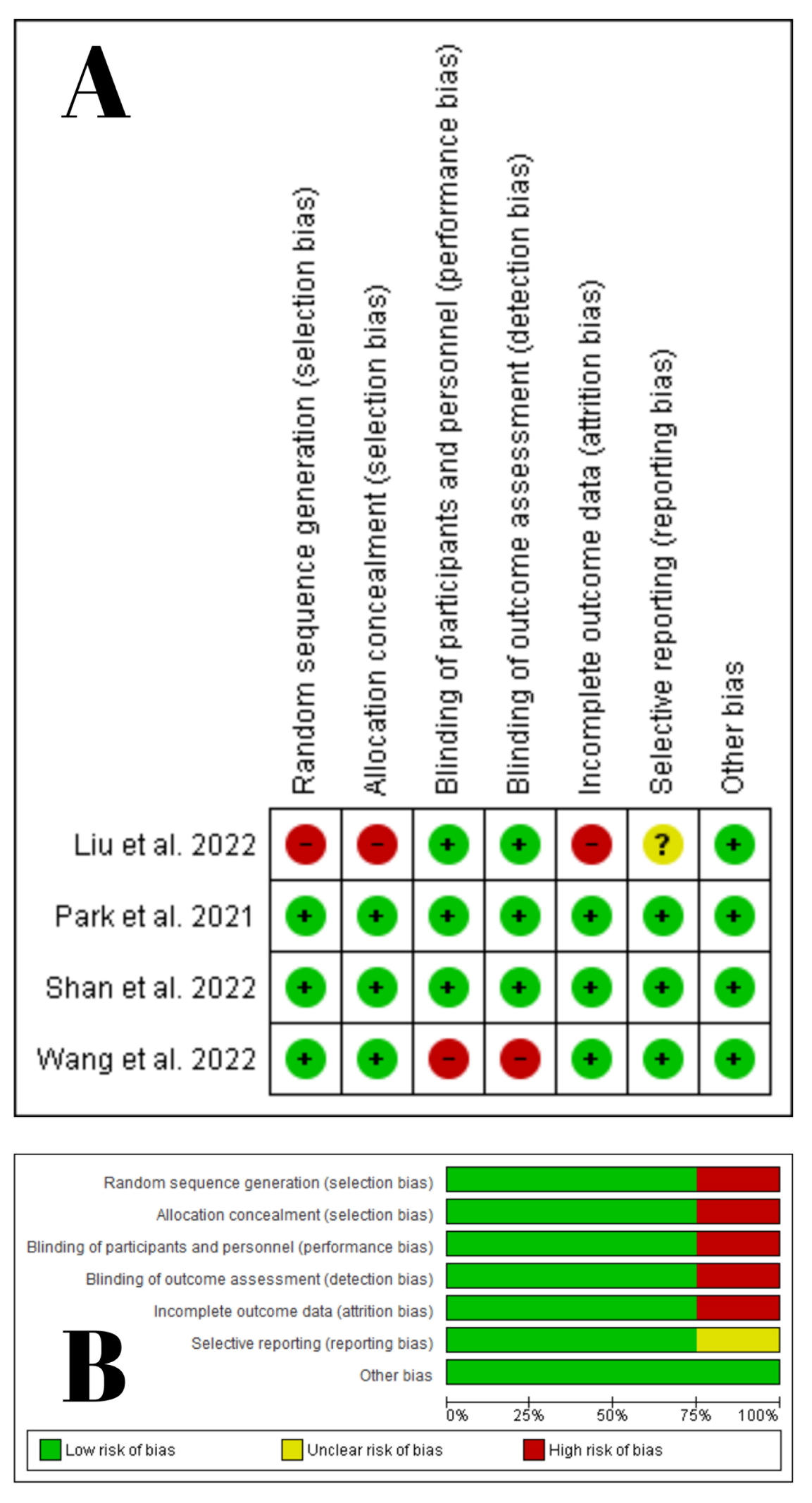
**



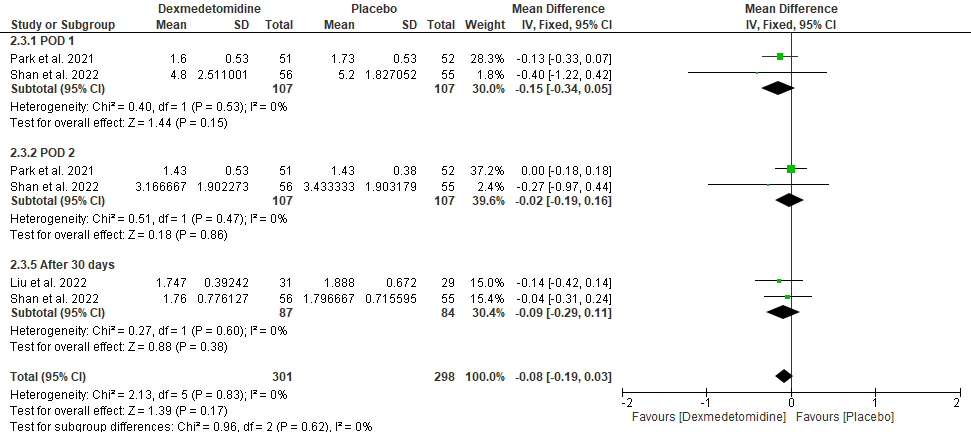


Figure S3: Forest plot of comparison: 2 Secondary Outcomes, outcome: 2.3 Cystatin C (mg/L).


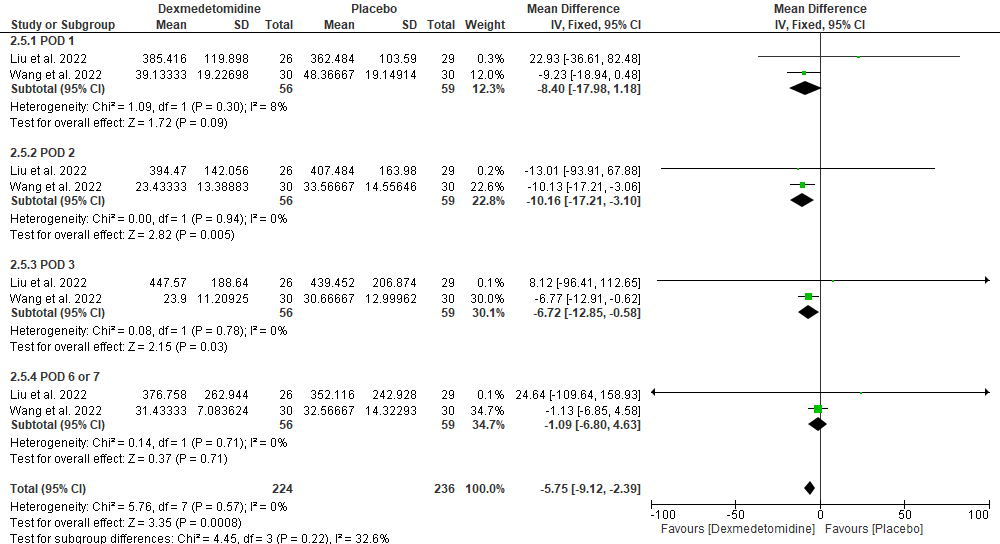


Figure S4: Forest plot of comparison: 2 Secondary Outcomes, outcome: 2.5 BUN (mg/dl).


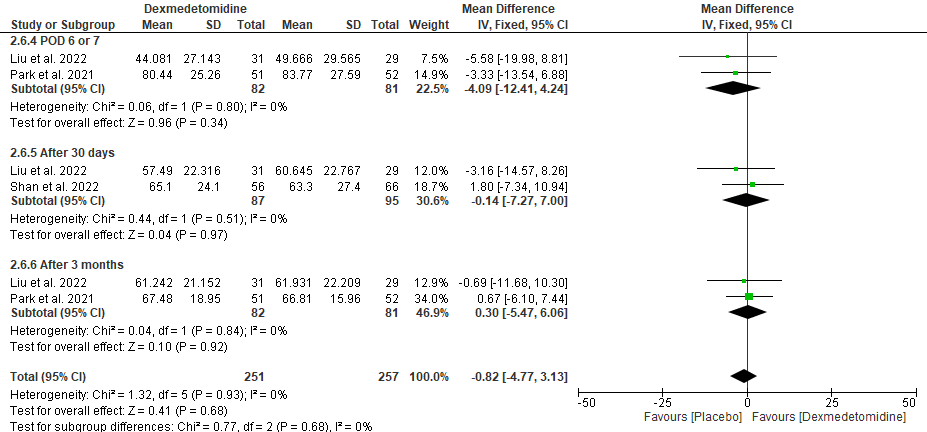


Figure S5: Forest plot of comparison: 2 Secondary Outcomes, outcome: 2.6 eGFR (mL/min/1.73 m2).
